# Water protection in patients with tympanostomy tubes in tympanic membrane: a randomized clinical trial

**DOI:** 10.31744/einstein_journal/2019AO4423

**Published:** 2019-02-25

**Authors:** Marcel Menon Miyake, Daniela Akemi Tateno, Natália Amaral Cançado, Michelle Menon Miyake, Stefano Tincani, Osmar Mesquita de Sousa

**Affiliations:** 1 Faculdade de Ciências Médicas, Santa Casa de São Paulo, São Paulo, SP, Brazil.; 2 Department of Otorhinolaryngology, Faculdade de Ciências Médicas, Santa Casa de São Paulo, São Paulo, SP, Brazil.

**Keywords:** Ear protective devices/utilization, Middle ear ventilation, Water/adverse effects, Postoperative complications/prevention & control, Otitis media with effusion/surgery, Dispositivos de proteção das orelhas/utilização, Ventilação da orelha média, Água/efeitos adversos, Complicações pós-operatórias/prevenção & controle, Otite média com derrame

## Abstract

**Objective:**

To analyze the incidence of otorrhea in the postoperative period of patients submitted to tympanotomy to place ventilation tube, and who did not protect the ear when exposed to water.

**Methods:**

Open, randomized-controlled trial. Eighty patients submitted to unilateral or bilateral ear grommet tympanostomy were included and divided into two groups: Auricular Protection and Non-Protection to water during bathing and activities in water.

**Results:**

In the first postoperative month, the Non-Protection Group presented a significant increase in the number of patients with otorrhea and in the incidence. Four patients of the Protection Group (11%) presented at least one episode of otorrhea in this period, representing an incidence of 0.11 (standard deviation ±0.32) episode/month, whereas in the Non-Protection Group there were 12 episodes (33%; p=0.045) and incidence of 0.33 (±0.48; p=0.02). Between the 2^nd^ and the 13^th^ postoperative months, there was no difference between groups. Seven patients in the Protection Group (20%) had at least one episode of otorrhea, representing an incidence of 0.04 (±0.09) episodes/month, while in the Non-Protection Group there were seven episodes (22%; p=0.8) and incidence of 0.05 (±0.1; p=0.8).

**Conclusion:**

Patients who underwent ear protection when exposed to water had a lower incidence of otorrhea in the first postoperative month than those who did not undergo protection. From the second month, there was no difference between groups.

## RESUMO

**Objetivo:**

Avaliar a incidência de otorreia no período pós-operatório em pacientes submetidos à timpanotomia para colocação de tubo de ventilação e que não realizaram proteção auricular quando expostos à água.

**Métodos:**

Ensaio clínico controlado, aberto e randomizado. Foram incluídos 80 pacientes submetidos à timpanotomia para colocação de tubo de ventilação unilateral ou bilateral, divididos em dois grupos: Grupo Proteção e Grupo Não Proteção auricular da água durante o banho e as atividades aquáticas.

**Resultados:**

No primeiro mês pós-operatório, o Grupo Não Proteção apresentou aumento significativo tanto no número de pacientes com otorreia quanto na incidência. Quatro pacientes do Grupo Proteção (11%) apresentaram ao menos um episódio de otorreia neste período, representando incidência de 0,11 (desvio padrão ±0,32) episódio/mês, enquanto no Grupo Não Proteção ocorreram 12 episódios (33%; p=0,045) e incidência de 0,33 (±0,48; p=0,02). Entre o 2º e o 13º meses pós-operatórios, não houve diferença entre os grupos. Sete pacientes do Grupo Proteção (20%) apresentaram ao menos um episódio de otorreia, representando incidência de 0,04 (±0,09) episódios/mês, enquanto no Grupo Não Proteção foram registrados sete episódios (22%; p=0,8) e incidência de 0,05 (±0,1; p=0,8).

**Conclusão:**

Pacientes que realizaram a proteção auricular quando expostos à água apresentaram menor incidência de otorreia no primeiro mês pós-operatório do que aqueles que não a realizaram. A partir do segundo mês, não houve diferença entre os grupos.

## INTRODUCTION

Tympanotomy for ventilation tubes placement is one of the most commonly performed procedures in children and adolescents worldwide. It is estimated that more than one million patients undergo surgery every year in the United States and Canada.^(^ ^1^ ^,^ ^2^ ^)^ One of the most common complications of this procedure is otorrhea, which can be a consequence of contamination of the middle ear by pathogens from two distinct regions: rhinopharynx, characterizing acute otitis media; and outer ear, by the transposition of the ventilation tube through its orifice.^(^ ^1^ ^,^ ^3^ ^)^

For many years, children with ventilation tubes were instructed to use ear protection when bathing, and not to engage in water activities, with the purpose of avoiding water entering the middle ear and preventing a possible infection. However, controlled studies and systematic reviews, carried out especially in the United States and Europe, concluded that children who ears were protected against water presented with otorrhea indices equal to or similar to those of children with exposed ears.^(^ ^3^ ^-^ ^6^ ^)^ Some experimental studies have also shown that the water traverses the ventilation tube only under pressure, or when it is mixed with substances that decrease its surface tension, such as soap.^(^ ^7^ ^,^ ^8^ ^)^ Thus, the precautions given would be unnecessary, decreasing the inconvenience and the deprivations caused in the everyday life of operated children.

Even so, a large part of physicians, especially in Brazil, continues reticent and recommends ear protection even during bathing. Inquiries made in the United States and Europe lead to the conclusion that, despite the evidence, ear protection continues to be recommended. However, most physicians allow water activities to be done, and half of them do not recommend any type of protection.^(^ ^9^ ^,^ ^10^ ^)^ In a quick questionnaire applied at the organization where this study was carried out, among the 30 assisting physicians who were interviewed, only two did not recommend ear protection during bathing, and all of them suggested avoiding water activities or sealing off the auditory canal with previously molded prostheses and a silicone cap.

In Brazil, no prospective, randomized and controlled study was identified when we searched the terms “water/ventilation tube” or “water/grommets” in the MEDLINE and LILACS databases.

## OBJECTIVE

To evaluate the incidence of postoperative otorrhea in patients submitted to tympanotomy for placement of ventilation tubes and who did not use ear protection when exposed to water.

## METHODS

A controlled open-label randomized clinical study was conducted, which included 80 patients from a sequential convenience sampling during the period between July 2013 and May 2015. The study was approved by the Research Ethics Committee, under opinion no. 299.403, dated 26 June 2013. The Informed Consent Form was signed by the patients themselves or by their caregivers. All patients presented with a diagnosis of otitis media with effusion and/or recurrent acute otitis media, having been submitted to placement of a unilateral or bilateral ventilation tube. Two different types of tubes were inserted (Sheppard or Armstrong), and this choice was determined by the surgeon. The sample was stratified by the number of ears operated on (unilaterally or bilaterally). Randomization was done in blocks of six patients each, in order to balance the number of patients among the groups. Surgical scheduling was made by physicians who did not participate in the process of patient enrolment in the study, thus avoiding induction of choice of one of the patient groups.

Surgical indications were based on the Clinical Practice Guideline: Tympanostomy Tubes in Children,^(^ ^1^ ^)^ which defined secretory otitis media as the presence of bilateral effusion in the middle ear for at least 3 months; and recurrent acute otitis media as three or more episodes of acute otitis media in the last 6 months, or four or more episodes in the last 12 months. Any immunocompromised patients were excluded (immunodeficiency syndromes, positive for AIDS or HIV, *diabetes mellitus* , current chemotherapy, or chronic use of systemic corticosteroid), craniofacial deformities, cleft palate, or who have undergone previous ear surgery (except for ventilation tube).

Patients were randomly divided into two groups: in the Protection Group, patients were instructed to use ear protection during bathing, with a cotton ball soaked in an oily solution or vaseline, and not to engage in water activities, or, if this was inevitable, to seal the ears with a pre-modeled ear protector and impermeable silicone cap. In the Non-Protection Group, patients were free to participate in any activity without ear protection. Both groups were instructed to avoid diving or any sudden movement in the water, swimming at great depths, and dunking one’s head in a bathtub with water and soap. At hospital discharge, all patients received a calendar on which any complication was to be reported: episodes in which they used no ear protection when exposed to water (Protection Group), swimming, infection of the upper airways, and otorrhea.

The follow-up visits were held one month after surgery, and then every three months until extrusion of the ventilation tubes. In case of otorrhea or any other ear-nose-throat complication, the patients were oriented to go the ENT emergency department of our organization.

Two primary outcomes were established: the number of patients that presented with at least one episode of otorrhea during the study period; and the incidence of otorrhea per patient (number of episodes de otorrhea/follow-up period). Such outcomes were compared between the Groups Protection and Non-Protection relative to the total follow-up time, at the first postoperative month, and at the 2^nd^ to 13^th^ postoperative month. Otorrhea associated with swimming was defined as any episode that occurred within seven days after this activity, and otorrhea associated with an upper respiratory tract infection (URTI) was characterized when it occurred within seven days after the onset of symptoms.

The statistical analysis of comparisons of number of patients with at least one episode de otorrhea between the groups was done with the χ^2^ test or Fischer’s exact test. Comparison between the incidences of otorrhea between the groups was conducted with the Mann-Whitney test. The demographic and patient follow-up characteristics were compared using the χ^2^ test for the categorical variables, and the Student’s *t* test for the continuous variables. To evaluate otorrhea as a function of the variables collected, the stepwise multiple logistic regression technique was used.

## RESULTS

**Figure 1:**
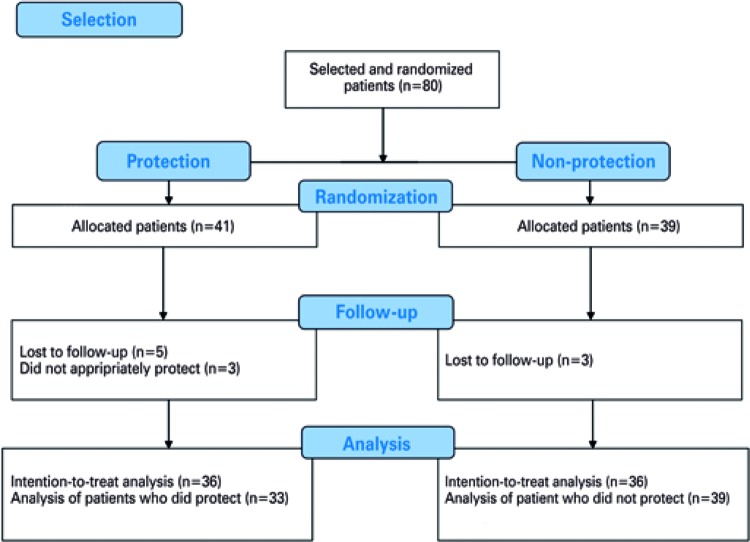
CONSORT study flowchart

A total of 80 patients were recruited by the study; 41 (51.2%) were randomly allocated to the Protection Group and 39 (49.8%) to Non-Protection Group ( Figure 1 ). The demographic and patient follow-up characteristics, and the distribution between the groups are shown on table 1 . The mean age (standard deviation) was 10.5 years (±15) in the Protection Group and 6.6 years (±5.5) in the Non-Protection Group.

**Table 1:** Demographic and follow-up characteristics of randomized patients

Demographic characteristics	Protection Group (n=41)	Non-protection Group (n=39)
Total number of patients	41	39
Age	10.5±15.0	6.6±5.5
Female sex	11 (27)	13 (33)
Race		
White	22 (54)	15 (38)
Brown	16 (39)	21 (54)
Black	3 (7)	3 (8)
Involvement		
Unilateral	8 (20)	8 (21)
Bilateral	33 (80)	31 (79)
Tube		
Sheppard	34 (82)	34 (87)
Armstrong	6 (16)	5 (13)
Sheppard + armstrong	1 (2)	0 (0)
Indication		
OME	31 (76)	29 (74)
rAOM	5 (12)	7 (18)
OME + rAOM	5 (12)	3 (8)
Otorrhea before the tube	15 (37)	17 (44)
Simultaneous adenoidectomy	36 (88)	31 (79)
Simultaneous tonsillectomy	30 (73)	21 (54)
Rhinitis	22 (54)	21 (54)
Bottle	14 (34)	15 (38)
Swimming	8 (20)	6 (15)
Active/passive smoking	9 (22)	17 (44)
Dust/moisture at home	14 (34)	10 (26)
Pets (dog, cat or bird)	22 (54)	16 (41)
Follow-up, months		
Did not return	5 (12)	3 (8)
1	1 (2)	5 (13)
4	8 (20)	11 (28)
7	9 (22)	5 (13)
10	12 (29)	7 (18)
13	6 (15)	8 (20)

Results expressed as n, mean±standard deviation, or n (%).

OME: otitis media with effusion; rAOM: recurrent acute otitis media.

Five patients (12%) from the Protection Group and three (8%) of the Non-Protection Group did not return for any follow-up visit. Among the patients who did return for at least one visit, the mean follow-up time of the Protection Group was 8.16 months (±3.3) and of the Non-Protection Group, it was 7.16 months (±4.2).

The comparison between the episodes and the incidence of otorrhea among patients who had been randomly divided into the Protection Group and Non-Protection Group, and who returned for at least one postoperative visit, is presented on table 2 . During the total follow-up period, there was no difference between the groups. Nine patients from the Protection Group (25%) presented with at least one otorrhea episode during the follow-up period, which is an incidence of 0.05 (±0.1) episode/month, while in the Non-Protection Group, 16 patients were recorded (44%) (p=0.08), with an incidence of 0.13 (±0.24; p=0.06). Similar results were observed between the 2^nd^ and 13^th^ month of follow-up. Seven patients from the Protection Group (20%) presented with at least one episode of otorrhea, with an incidence of 0.04 (±0.09) episode/month, whereas in the Non-Protection Group, seven episodes were found (22%; p=0.8) with an incidence of 0.05 (±0.1, standard deviation; p=0.8). When the comparison was made only in the first postoperative month, the Non-Protection Group showed a significant increase both in number of patients with otorrhea and in incidence. Four patients from the Protection Group (11%) presented with at least one episode of otorrhea during this period, with an incidence of 0.11 (±0.32) episode/month, while in the Non-Protection Group, there were twelve episodes (33%; p=0.045) with an incidence of 0.33 (±0.48; p=0.02).

**Table 2:** Presence and incidence of otorrhea per group

	Protection Group (n=36)	Non-Protection Group (n=36)	p value
Patients with at least one episode of otorrhea			
Total period	9 (25)	16 (44)	0.08
Month 1	4 (11)	12 (33)	0.045
Months 2 and 13	7 (20)	7 (22)	0.8
Incidence of otorrhea*			
Total period	0.05 (0.1)	0.13 (0.24)	0.06
Month 1	0.11 (0.32)	0.33 (0.48)	0.02
Months 2 and 13	0.04 (0.09)	0.05 (0.1)	0.8

Results expressed as n (%) or mean (± standard deviation). * Episodes of otorrhea/month.

Among the 36 patients randomized to the Protection Group who returned for at least one postoperative visit, three reported having used insufficient or no protection. In this way, with the purpose of comparing the association between the intervention (protection/non-protection) and the primary outcome (otorrhea), the 33 patients from the Protection Group, who in fact did protect their ears, with 39 patients who did not protect them (36 from the Non-Protection Group + 3 from the Protection Group who did not comply with the intervention proposed) ( Table 3 ).

**Table 3:** Presence and incidence of otorrhea per use of protection

	Protection Group (n=33)	Non-Protection Group (n=39)	p value
Patients with at least one episode of otorrhea			
Total period	7 (21)	18 (46)	0.04
Month 1	4 (12)	12 (31)	0.08
Months 2 and 13	5 (16)	9 (25)	0.34
Incidence of otorrhea*			
Total period	0.04 (0.1)	0.13 (0.23)	0.03
Month 1	0.12 (0.33)	0.30 (0.46)	0.06
Months 2 and 13	0.03 (0.08)	0.05 (0.11)	0.32

Results expressed as n (%) or mean (± standard deviation). * Episodes of otorrhea/month.

During the total follow-up period, a greater number of patients who did not protect their ears from exposure to water presented with otorrhea (p=0.04), just as they had a higher incidence of otorrhea (p=0.03), when compared to those who did, in fact, protect them. Considering only the first month of follow-up, a tendency was noted towards a greater number of patients with otorrhea among those who did not protect their ears (p=0.08), as well as in the incidence (p=0.06). There was no difference in the comparison between the groups between the 2^nd^ and 13^th^ months as to number of patients with otorrhea (p=0.34) and incidence of otorrhea (p=0.32).

There were 13 reports of swimming and 18 of URTI in the Protection Group, with no associated otorrhea. In the Non-Protection Group, ten reports of swimming were made (with two associated episodes of otorrhea) and 22 of URTI (with seven episodes of associated otorrhea). The logistic regression analysis determined as risk factors for the development of otorrhea, the preoperative diagnoses of recurrent otitis media (p=0.02; odds ratio − OR: 1.23-19.22) and recurrent otitis media associated with otitis media with effusion (p=0.02; OR: 1.49-12.81), relative to the isolated diagnosis of otitis media with effusion. No other variable presented with a significant difference.

## DISCUSSION

This study is the first controlled and randomized clinical trial carried out in Brazil that evaluated the need of patients submitted to ventilation tube placement in the tympanic membrane for protecting their ears when exposed to water. Our results showed that, as of the second postoperative month, protecting the ears when exposed to water did not represent the lowest number of patients with at least one episode of otorrhea (20% of patients from the Protection Group *versus* 22% of the Non-Protection Group; p=0.8), nor the lowest incidence of otorrhea (0.04±0.05 in the Protection Group *versus* 0.05±0.1 in the Non-Protection Group). Similar results were observed when the comparison was made between patients randomized to the Protection Group who, in fact, did comply with the proposed management and the patients who did not.

These results are in accordance with prior publications that concluded children avoiding ear exposure to water presented with infection rates equal or similar to children exposed. The meta-analyses carried out by Lee et al._,_^(^ ^4^ ^)^ (619 children from 5 studies) and Carbonell et al.,^(^ ^5^ ^)^ (943 patients from 11 studies) agreed that there is no difference relative to the rates of otorrhea between patients who did not protect their ears when swimming as compared to those who did protect them or who did not swim. Nevertheless, the studies compiled by these reviews showed problems with the study design, absence of randomization, blinding, compliance, or small samples.^(^ ^11^ ^,^ ^12^ ^)^ The only clinical study, randomized and single-blind, with evidence level 1^(^ ^3^ ^)^ observed a minimal increase in the rate of otorrhea in patients who did not use ear protection when exposed to water. The difference observed between the groups was 0.36 infection per year/child, which means that one child would need to protect the ears for 2.8 years to prevent one episode of otorrhea. Despite this difference, the use of ear protectors can be exempted in the routine of these patients.^(^ ^3^ ^,^ ^12^ ^)^

On the other hand, during the first postoperative month, ear protection when exposed to water resulted in a lower incidence (0.11±0.32 in the Protection Group *versus* 0.33±0.48 in the Non-Protection Group; p=0.02), and a smaller number of patients with otorrhea (11% of patients from the Protection Group *versus* 33% of the Non-Protection Group; p=0.045). Similar results were noted when comparing patients randomized to the Protection Group who complied with the proposed management and the patients who did not.

The incidence of otorrhea in patients not using ear protection when exposed to water in the first postoperative month was rarely studied previously, since most studies initiated patient assessment at least two weeks after surgery.^(^ ^3^ ^,^ ^6^ ^)^ This study presented the highest incidence and most patients with episodes of otorrhea, possibly by persistence, in the immediate postoperative period, of peripheral openings on the ventilation tube, resulting from an ample myringotomy, which allows the inflow of water into the middle ear, causing infection. With the healing of the tympanic membrane, these openings should close, and the orifice of the ventilation tube should remain as the only communication between the outer and middle ear, which would justify the decrease in late postoperative infection rates (as of the second month).

This investigation presented with some limitations. Due to the criteria established for the association between otorrhea and swimming or URTI, the small number of patients who swam, and the effusion culture not having been performed, it was not possible to precisely distinguish the cases that originated in the middle ear from those that began in the outer ear.

The decision between protecting the ear or not when exposed to water should be individualized and discussed among the physician, the patient, and family members. The results obtained in this study, associated with observations from previous studies, allow us to conclude that it is safe to instruct patients with tympanic ventilation tubes not to protect their ears during bathing after the first postoperative month. Protection should be recommended during the first month after surgery and in cases of recurrent otorrhea or discomfort in the ear when exposed to water. With the small number of patients who swam during the study period, it was not possible to conclude, either, if it is safe to swim without any protection. However, based on the literature available,^(^ ^3^ ^,^ ^4^ ^,^ ^11^ ^,^ ^12^ ^)^ to allow water surface activities with no ear protection seems to present a minimum risk. Deep diving or bathing in a bathtub with soap should be avoided, or ear protection should be recommended. To prohibit patients from swimming is an unnecessary procedure, and especially in children, hinders leisure times and the development of swimming and self-protection skills in water.

## CONCLUSION

Patients who protected and who did not protect their ears when exposed to water after tympanotomy for ventilation tube placement, presented with a similar incidence of otorrhea episodes as of the second postoperative month. In the same way, as of the second postoperative month, the number of patients who presented with at least one episode of otorrhea was similar in both groups.
